# BAP1 as a predictive biomarker of therapeutic response to oncolytic vaccinia virus for metastatic renal cell carcinoma therapy

**DOI:** 10.1007/s00262-025-04139-4

**Published:** 2025-08-06

**Authors:** Jee Soo Park, Won Sik Jang, Myung Eun Lee, Jongchan Kim, Keunhee Oh, Namhee Lee, Won Sik Ham

**Affiliations:** 1 Department of Urology and Urological Science Institute, Yonsei University College of Medicine, Seoul, 03722 Republic of Korea; 2 Department of Urology, Yongin Severance Hospital, Yonsei University Health System, Yongin, Republic of Korea; 3 Research Center, SillaJen, Inc., Seoul, Republic of Korea

**Keywords:** BAP1, Oncolytic vaccinia virus, Predictive biomarker, Pexastimogene devacirepvec (pexa-vec), Renal cell carcinoma

## Abstract

**Background:**

The therapeutic efficacy of oncolytic vaccinia virus (JX-594) has been demonstrated in metastatic clear cell renal cell carcinoma (ccRCC); however, only selected patients respond, and there are no predictive biomarkers for therapeutic response. We aimed to identify predictive biomarkers for JX-594 treatment and elucidate the underlying mechanisms.

**Methods:**

Four cell line-derived xenograft (CDX) models were developed using representative ccRCC cell lines harboring common mutations. Tumors were subcutaneously implanted into the right flank of BALB/c nude mice. Mice were treated with vehicle or JX-594 via intratumoral injection on days 0, 3, and 6, and tumor growth was evaluated. Therapeutic efficacies of JX-594 and a STING agonist were compared in the BAP1-mutant (769-P) CDX model.

**Results:**

All four CDX models showed significant tumor shrinkage following JX-594 treatment versus control. JX-594 exhibited greater efficacy than the STING agonist in BAP1-deficient xenografts. The BAP1 mutation was associated with rapid tumor progression and a stronger response to JX-594. JX-594 induced IFN-β expression through IRF7-dependent signaling in BAP1-deficient cells, bypassing impaired STING–IRF3 signaling.

**Conclusions:**

We identified BAP1 as a potential predictive biomarker for JX-594 treatment and explored its underlying mechanisms. However, given that the study used immunodeficient models, the findings reflect tumor-intrinsic interferon responses and require further validation in immunocompetent models to assess immune microenvironment modulation and clinical relevance.

**Supplementary Information:**

The online version contains supplementary material available at 10.1007/s00262-025-04139-4.

## Background

Renal cell carcinoma (RCC) accounts for 90% of the renal mass, with clear cell RCC (ccRCC) being the most common histological subtype [1]. Approximately 33% of the patients with RCC exhibit distant metastasis at diagnosis, and 20–30% of the patients report recurrence following nephrectomy [2]. Although advanced RCC is associated with poor prognosis, advances in systemic therapies, including immuno-oncology (IO) agents, have improved the oncological outcomes of RCC [1, 3]. However, only 20–30% of the patients respond to immune checkpoint inhibitor (ICI) treatment and the non-inflamed “cold” tumor microenvironment (TME) causes intrinsic resistance to ICI treatment [4]. To overcome this limitation, novel IO agents focus on gearing the TME toward immune-stimulating and tumor-suppressive phenotypes [5]. Owing to its ability to remodel the TME toward a T cell-inflamed phenotype, which translates to a “hot” TME [4], the oncolytic virus (OV) is a promising agent for this treatment strategy [6]. We previously reported the therapeutic efficacy of the oncolytic vaccinia virus, following the systemic injection of JX-594 (pexastimogene devacirepvec, Pexa-vec) in a metastatic (m)RCC murine model [7]. Despite the failure of the phase III PHOCUS trial in hepatocellular carcinoma (HCC), which likely stemmed from suboptimal patient selection and lack of biomarker guidance, recent clinical trials have explored Pexa-Vec in various cancer types, including RCC (NCT03294083), melanoma (NCT04849260), and prostate cancer (EudraCT 2012–000704-15). Furthermore, combination trials with immune checkpoint inhibitors are underway, while next-generation candidates like JX-970 are under development. This context underscores the potential relevance of the vaccinia virus platform and the need for biomarker-driven strategies to optimize patient selection.

Despite the development of numerous systemic agents for mRCC, such as targeted therapies and ICIs [8], there are no predictive biomarkers that facilitate the optimization of the sequences of current treatments [9]. Personalized treatment strategies for mRCC that integrate evolutionary trajectories and genomic characterization are urgently needed; however, molecular profiling of RCC is not currently used in routine clinical care [9]. Numerous studies have reported the usefulness of genetic mutation status as a predictive biomarker for systemic treatments [9–11]. For example, tumors associated with the *PBRM1* mutation respond favorably to angiogenic therapies, whereas those associated with a *BAP1* mutation respond poorly to the same treatment [10, 11]. The *PBRM1* mutation and sarcomatoid/rhaboid features are predictive biomarkers for ICI treatment [12, 13].

The first meta-analysis of previously published genomic data on ccRCC tumors demonstrated that *VHL, PBRM1, SETD2,* and *BAP1* are the four most commonly mutated genes in ccRCC [14]. In this study, we evaluated the therapeutic efficacy of JX-594 in vitro and in vivo based on the four most commonly mutated genes in ccRCC and investigated the underlying mechanisms.

## Methods

### ccRCC cell lines

Representative ccRCC cell lines, with genes that are commonly mutated, were selected; 786-O (cat# CRL-1932) represents ccRCC cells with a *VHL* mutation, and Caki-2 (cat# HTB-47) ccRCC cells with *VHL* and *PBRM1* mutations. A-498 (cat# HTB-44) represents ccRCC cells with *VHL* and *SETD2* mutations, and 769-P (cat# CRL-1933) represents ccRCC cells with *VHL* and *BAP1* mutations [15]. The human RCC cell lines were purchased from the American Type Culture Collection (Manassas, VA, USA) and cultured in Roswell Park Memorial Institute (RPMI)-1640, Eagles Minimum Essential Medium (EMEM), or McCoy’s 5A medium supplemented with 10% fetal bovine serum (Gibco; Thermo Fisher Scientific, Waltham, MA, USA) and 1% penicillin–streptomycin (Sigma-Aldrich, St. Louis, MO, USA) at 37 °C in a humidified atmosphere containing 5% CO_2_.

### Development of cell-line derived xenograft (CDX) mouse models

Animal experiments were conducted in accordance with the Guide to the Care and Use of Laboratory Animals approved by the Association for Assessment and Accreditation of Laboratory Animal Care and the National Institutes of Health. The experimental protocol was approved by the Institutional Animal Care and Use Committee (IACUC) of the Yonsei University Health System (IACUC No. 2020–0006) and followed the guidelines specified by the Institute of Laboratory Animal Resources Commission on Life Sciences National Research Council in the USA.

**Fig. 1:**
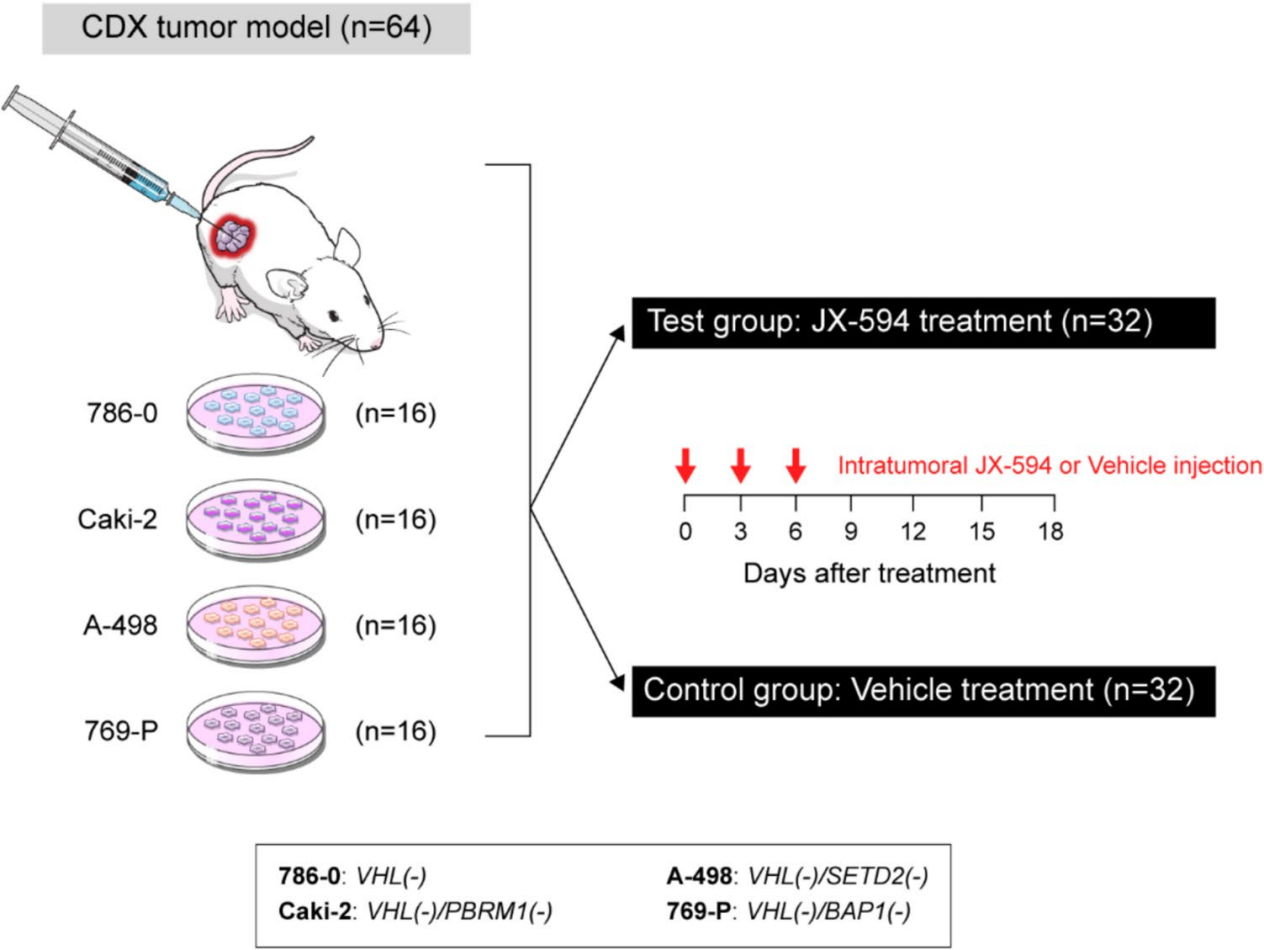
Development of cell-line derived xenograft (CDX) tumor models. Tumor models were developed based on four of the most commonly mutated genes (*VHL, PBRM1, SETD2,* and *BAP1*) in clear cell renal cell carcinomas (ccRCC). Tumor cells were subcutaneously injected in the right flank of mice. When the tumor volume exceeded 50 mm.^3^ (day 0), mice were subjected to vehicle or JX-594 via intratumoral injection on days 0, 3, and 6

In total, 64 adult BALB/c nude mice (Orient Bio Inc., Seongnam, Gyeonggi-Do, Korea) aged 6–7 weeks were maintained in clean animal facilities at the Yonsei University Health System. Four human RCC cell lines, selected according to the most commonly mutated genes (*VHL, PBRM1, SETD2*, and *BAP1*), were subcutaneously injected (2 × 10^6^ cells) into the right flank of the mice to implant tumors. Of the 64 mice, 32 accounted for the test group treated with JX-594 while the remaining 32 represented the control group treated with vehicle. In both groups, eight mice were allocated to each RCC cell line. After day 18 of injection, the mice were euthanized, and their tumor tissues were harvested (Fig. 1).

### Treatments and measurements of CDX models

JX-594, provided by SillaJen Inc. (Seoul, Korea), is a Wyeth strain vaccinia that was modified with human granulocyte macrophage colony-stimulating factor and Lac-Z genes inserted into the vaccinia thymidine kinase gene region under the control of the synthetic early and late promoter and p7.5 promoter, respectively [16].

When tumor volumes exceeded 50 mm^3^, either vehicle or 1 × 10^7^ plaque-forming units of JX-594 were intratumorally injected into the mice on days 0, 3, and 6 (Fig. 1). In addition, 3 mg/kg of a stimulator of interferon genes (STING) agonist (diABZI) was intratumorally injected on days 0, 3, and 6 for direct comparison; *BAP1*-deficient xenografts were used to compare the therapeutic efficacy of JX-594 and the STING agonist. Tumor sizes were measured using a digital caliper every 3 d, and tumor volumes were calculated using the modified ellipsoid formula [1/2 × (length × width^2^)]. Tumor growth rate was calculated as the increase in tumor volume divided by the number of days, which ranged between 0 and 12. The JX-594-induced decrease in tumor volume was calculated as the decrease in tumor volume from days 3 through 12. The relative therapeutic response ratio was calculated using the following formula: (decrease in tumor volume × tumor growth rate) to adjust for the relative differences in the tumor growth rate of each cell line and to compare the therapeutic effects of JX-594 in each cell line.

### Interferon beta (IFN-β) expression according to genetic mutations in ccRCC cell lines

The mRNA expression levels of interferon beta (IFN-β) in the four most commonly mutated ccRCC cell lines were measured using qRT-PCR after JX-594 and control treatment. Total RNA was extracted from cells using TRIzol reagent (Invitrogen, Carlsbad, CA, USA) and reverse-transcribed into first-strand cDNA using a Maxime RT-PCR PreMix Kit (iNtRON Bio-technology, Seongnam, Gyeonggi-Do, Korea) according to the manufacturer's protocol. qRT-PCR was performed using Power SYBR® Green Master Mix (Applied Biosystems, Foster City, CA, USA) in a 10 μL reaction comprising 5 μL SYBR® Green Master PCR mix, 1 μL of each forward and reverse primer (10 pmol), 1 μL diluted cDNA template, and sterile distilled water. The primer sequences are listed in Supplementary Table 1. Conditions for amplification were as follows: initial denaturation at 95 °C for 10 min; 40 cycles of denaturation at 95 °C for 15 s, annealing at 58 °C for 30 s, and elongation at 72 °C for 30 s; final elongation at 72 °C for 5 min. qRT-PCR was performed using an ABI StepOnePlus Real-Time PCR System (Applied Biosystems). Data were normalized to *GAPDH* expression, and relative gene expression was analyzed using the 2^−ΔΔCT^ method. The qRT-PCR experiments were repeated at least thrice, and the results were analyzed by a blinded investigator.

### Measurement of STING, interferon regulatory factor (IRF) 3, and IRF7 expression after STING agonist and/or JX-594 treatments according to genetic mutations in ccRCC cell lines

Cells were treated with 1 MOI JX-594 (SillaJen Inc., Seoul, Korea) and/or 1 μM diABZI (Invivogen, San Diego, CA, USA), a STING agonist, for 24 h. Cells were lysed with RIPA buffer, and the resultant proteins separated on sodium dodecyl sulfate–polyacrylamide gels and probed using the following primary antibodies: anti-STING (#13647S, Cell Signaling Technology, Danvers, MA, USA), anti-phospho-IRF3 (#37829S, Cell Signaling Technology), anti-IRF3 (#33641S, Cell Signaling Technology), anti-phospho-IRF7 (#5184S, Cell Signaling Technology), anti-IRF7 (#74472S, Cell Signaling Technology), and anti-β-actin (#47,778, Santa Cruz Biotechnology, Dallas, TX, USA). They were subsequently incubated with horse radish peroxidase-conjugated secondary antibodies (Cell Signaling Technology). To detect the reactive bands, the membranes were examined using the ECL Prime Western Blotting Detection System (GE Healthcare, Amersham, UK) and LAS-3000 (Fujifilm, Tokyo, Japan).

### Statistical analysis

Statistical analyses were performed using GraphPad Prism version 8.0 (GraphPad Software, Inc., La Jolla, CA, USA) and SPSS version 23.0 (IBM Corp., Armonk, NY, USA). All results are expressed as the mean ± standard deviation, unless otherwise indicated. Student’s *t*-tests were conducted, except for the datasets that did not follow a normal distribution based on the Shapiro–Wilk normality test. If the dataset did not follow a normal distribution, the Mann–Whitney U test was used. All statistical tests were two-tailed, and statistical significance was set at *p* < 0.05.

## Results

### Comparison of the therapeutic efficacy of JX-594 according to genetic mutations in CDX models

A significant decrease was observed in the tumor sizes of all four CDX models from day 9 after JX-594 treatment compared to vehicle treatment.

**Fig. 2:**
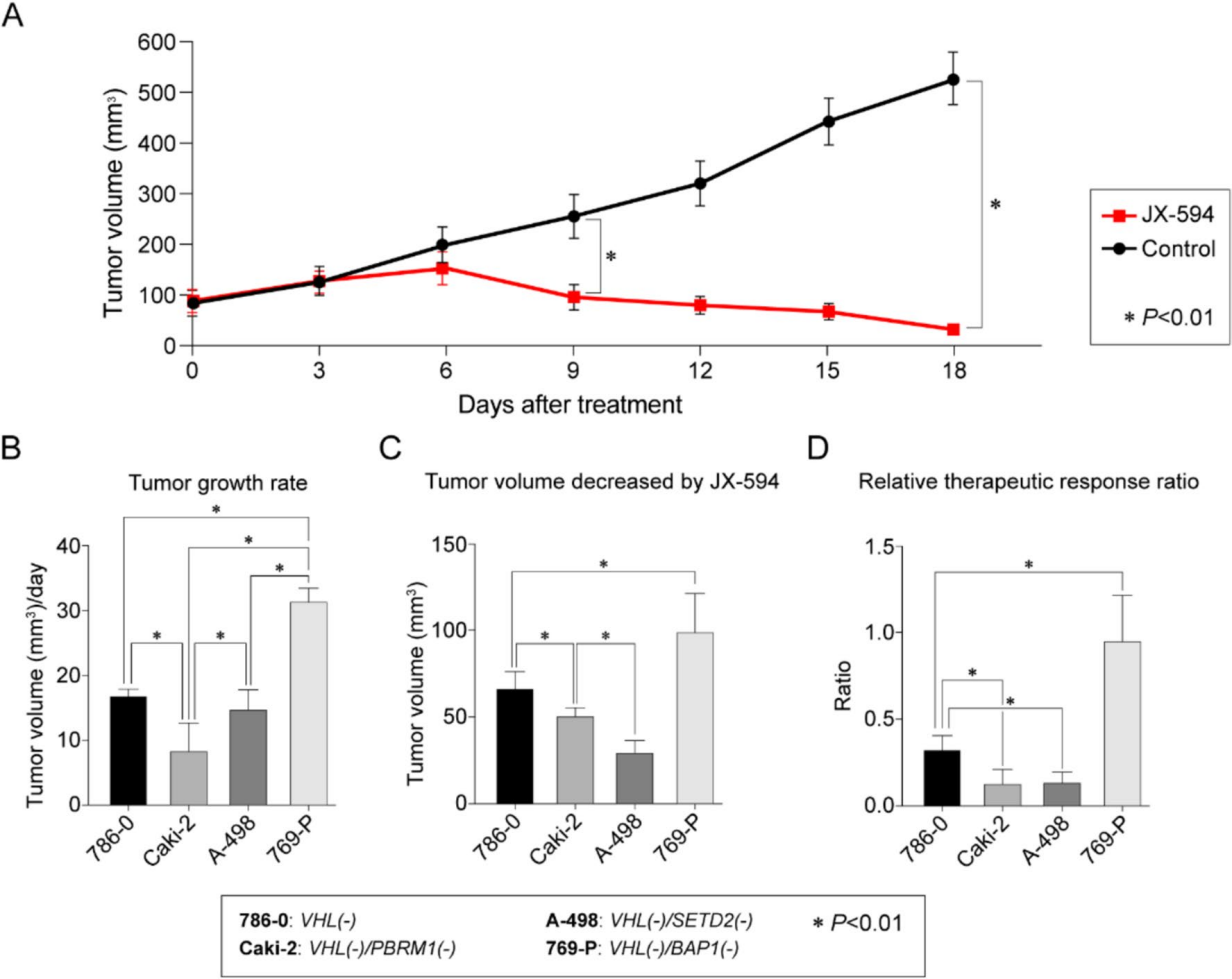
Evaluation of CDX tumor models treated with JX-594 or control (vehicle). **a** Growth curves of JX-594-treated and control groups. Bar graphs representing **b** tumor growth rate in the control group, **c** decrease in tumor volume for the JX-594-treated group, and **d** relative therapeutic response ratio (decrease in tumor volume × tumor growth rate) of tumors with *VHL, PBRM1, SETD2,* and *BAP1* mutations following treatment with JX-594

For subgroup analysis by genetic mutations, the CDX model with *BAP1* mutations exhibited the most rapid tumor growth rate, compared with the models with *VHL*, *PBRM1*, and *SETD2* mutations (*P* < *0.01*; Fig. 2a). The CDX model with *PBRM1* mutations had the lowest tumor growth rate compared to that of models with other genetic mutations (*P* < 0.01; Fig. 2a).

The JX-594-induced decrease in tumor volume was the most significant in tumors associated with *BAP1* mutations as opposed to tumors with *VHL, PBRM1,* and *SETD2* mutations (*P* < *0.01*; Fig. 2b). In contrast, tumors with *SETD2* mutations demonstrated the least reduction in volume following JX-594 treatment compared with tumors with other mutations (*P* < *0.01*; Fig. 2b).

Tumors with *BAP1* mutations exhibited a significantly more optimized therapeutic ratio—which was adjusted for the relative difference in the tumor growth rate of each cell line—than tumors with other mutations. Tumors with *VHL* mutations ranked second with respect to the therapeutic index following JX-594 treatment. Furthermore, no statistically significant difference (*P* = *0.86)* was observed between the therapeutic ratios of tumors with *PBRM1* and *SETD2* mutations.

### Comparison of IFN-β expression levels based on genetic mutations in ccRCC cell lines after JX-594 treatment

**Fig. 3:**
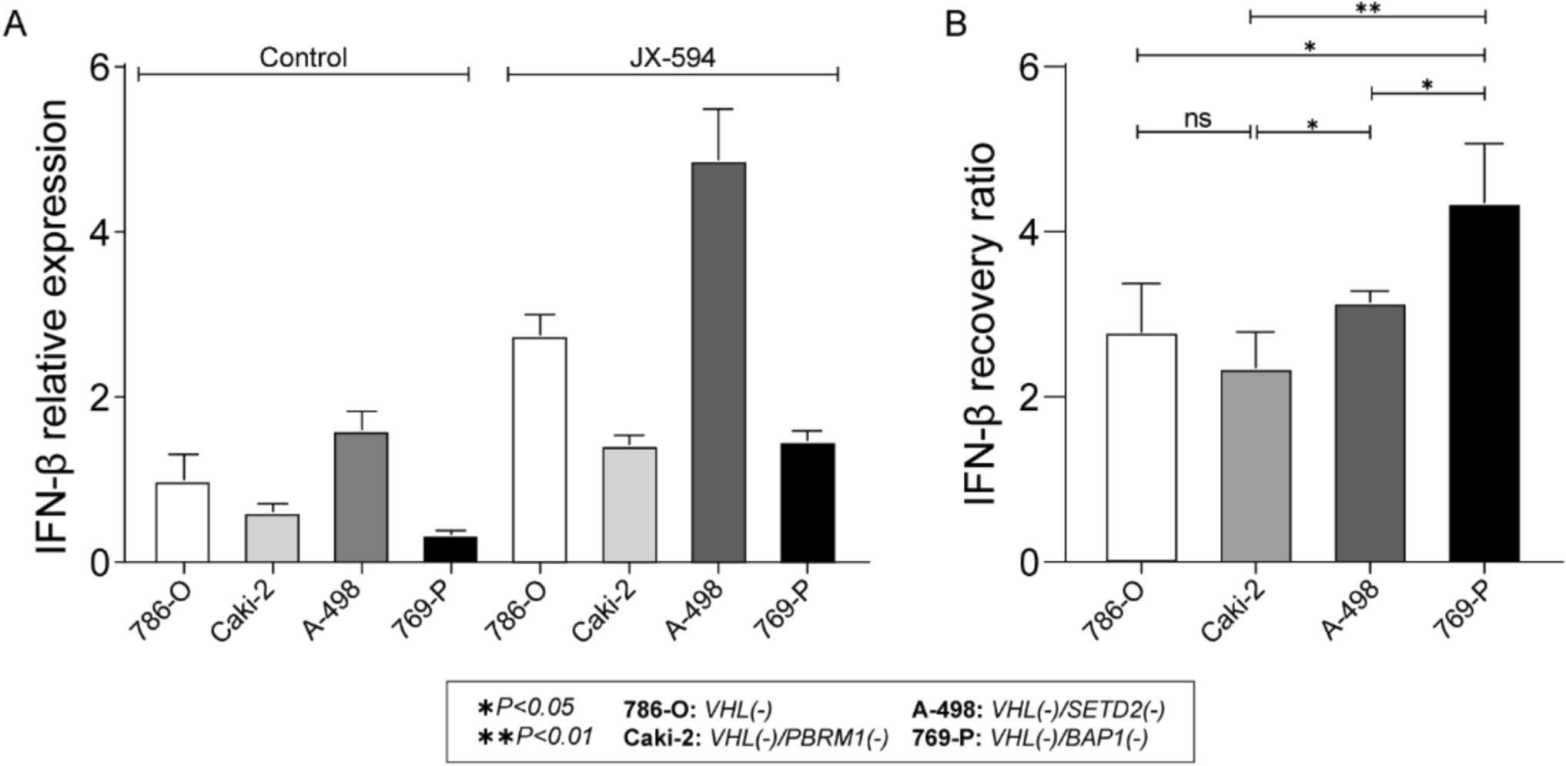
IFN-β mRNA expression in ccRCC cell lines with four of the most commonly mutated genes. **a** Measurement of IFN-β expression levels in JX-594-treated and control (vehicle) mice. **b** IFN-β recovery ratio of ccRCC cell lines, calculated as IFN-β levels after JX-594 treatment divided by control (vehicle) treatment

The mRNA expression of IFN-β in the four most commonly mutated ccRCC cell lines after treatment with JX-594 or vehicle was evaluated. IFN-β expression was significantly decreased in the *BAP1*-deficient cell line compared with that in the other cell lines in the control and treatment groups (Fig. 3a). The IFN-β levels generally increased in all cell lines after JX-594 treatment, and the *BAP1*-deficient cell line had the highest recovery rate (Fig. 3b).

### STING, IRF3, and IRF7 expression levels after JX-594 and/or STING agonist treatment according to genetic mutations in ccRCC cell lines

**Fig. 4:**
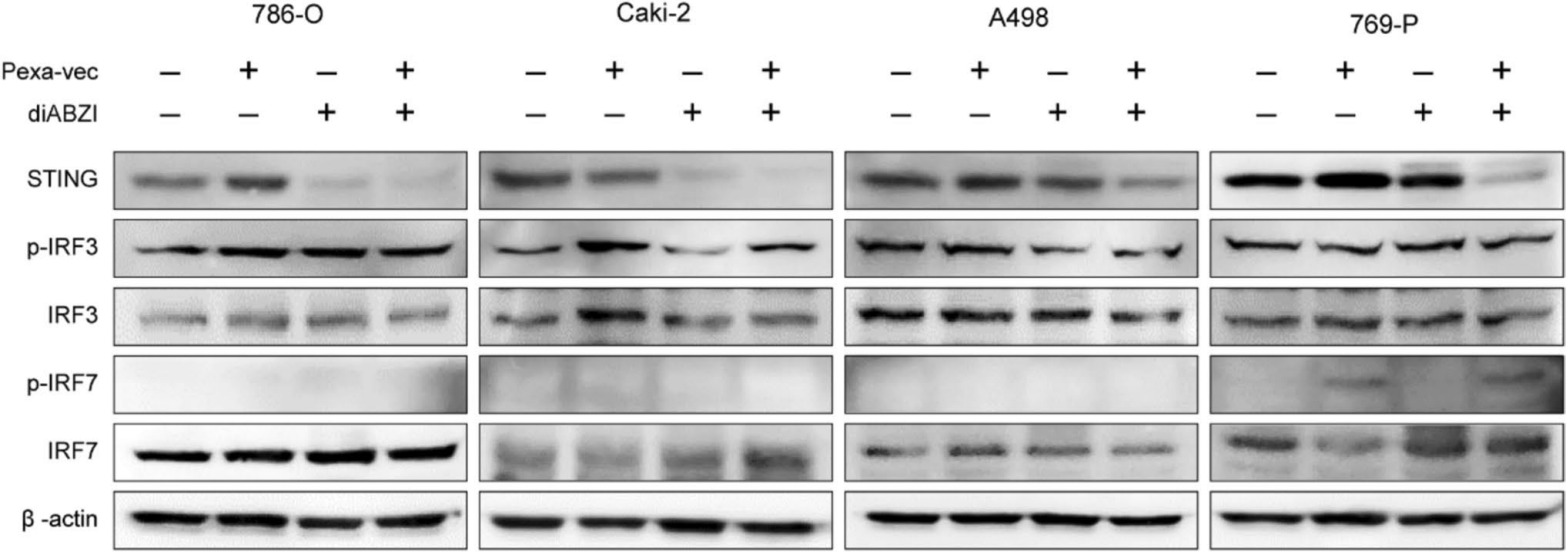
Sodium dodecyl sulfate-solubilized whole cell lysates of 786-O, Caki-2, A-498, and 769-P cell lines (n = 3) treated for 24 h with 1 MOI JX-594 and/or 1 μM diABZI and probed with the indicated antibodies

Cells were treated with JX-594 and/or diABZI and the protein levels of STING, IRF3, phosphorylated IRF3, IRF7, and phosphorylated IRF7 were measured (Fig. 4). Following treatment with diABZI, the protein levels of STING in the 786-O and Caki-2 cell lines markedly decreased, but this decrease was only slight in the A-498 cell line. The protein levels of STING in 769-P cells treated with diABZI exhibited no reduction but decreased markedly following treatment with a combination of JX-594 and diABZI. The protein levels of phosphorylated IRF3 were similar after JX-594 and/or diABZI treatments in the 786-O cell line. However, JX-594 treatment significantly increased the levels of phosphorylated IRF3 compared with the diABZI treatment in the Caki-2 cell line. There were no significant differences between the levels of phosphorylated IRF3 for A-498 and 769-P cells treated with JX-594 and/or diABZI. The levels of phosphorylated IRF7 increased only in 769-P cells treated with JX-594.

### Comparison of therapeutic efficacy of JX-594 or STING agonist in BAP1-deficient xenograft models

**Fig. 5:**
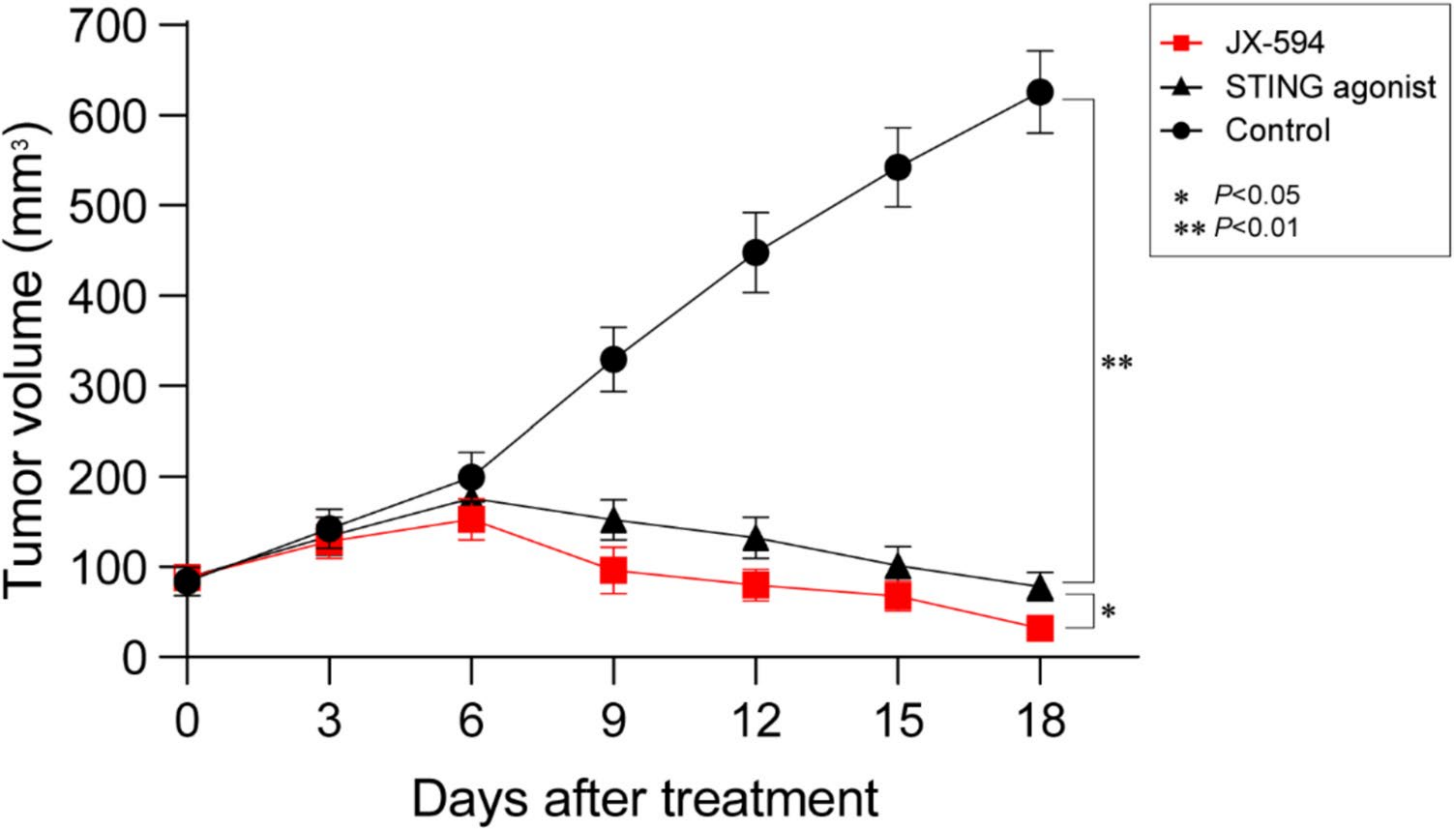
Growth curves of the 769-P CDX tumor models. The tumor treated with JX-594, STING agonist (diABZI), or control (vehicle)

*BAP1*-deficient xenograft models demonstrated a significant decrease in tumor size following JX-594 or diABZI treatment compared to vehicle treatment from day 9 (Fig. 5). Although JX-594 and diABZI treatments significantly decreased the tumor size, compared with the control, JX-594 treatment significantly reduced the tumor size compared with diABZI treatment on day 18 (*P* < 0.05; Fig. 5).

## Discussion

Our study demonstrated that the *BAP1* mutation is associated with rapid tumor progression and the therapeutic responses to JX-594 were most pronounced in cells with *BAP1* mutations. Hence, the *BAP1* mutation was identified as a predictive biomarker for JX-594 treatment through IRF7-dependent pathways.

In 2018, the Tracking Cancer Evolution through therapy (TRACERx) renal consortium published a study on the origin, evolution, and routes to metastatic ccRCCs [17–19]. We selected the target gene for this study according to the evolutionary lineage of the TRACERx renal cohort [18]. Recently, Bui et al. published the first meta-analysis of genomic data from ccRCC tumors, reporting the four most commonly mutated genes in ccRCC–*VHL, PBRM1, SETD2,* and *BAP1*–with mutation prevalences of 64%, 36%, 20%, and 13%, respectively [14]. *VHL* is a tumor suppressor gene located on chromosome 3p and is associated with hypoxia-inducible factors, which serve as potent transcriptional proangiogenic factors in RCC carcinogenesis [20]. The three other most frequently mutated genes, *PBRM1, SETD2,* and *BAP1,* are also located on chromosome 3p and contribute to DNA repair and transcriptional activation [14].

*PBRM1* and *BAP1* mutations are mutually exclusive. *BAP1*-mutant tumors are associated with poorer prognosis and a higher Fuhrman grade than *PBRM1*-mutant tumors [21, 22]. This is consistent with the MSKCC cohort study findings, where *BAP1* mutations were associated with poor prognostic factors, such as a higher T stage, higher nuclear grade, larger tumor size, more necrosis, and the presence of metastatic disease at presentation [23]. Our previous study also confirmed that *BAP1* expression levels significantly decreased in aggressive T1 stage ccRCC compared with those in non-aggressive T1 stage ccRCC [24]. In this study, tumors with *BAP1* mutations exhibited the most rapid growth rate when compared to those with other mutations.

Furthermore, the therapeutic response was the most effective in case of *BAP1-*mutant tumors even after the relative tumor growth rate was adjusted, which may be attributed to the mechanism of action of OVs. Among their two primary therapeutic mechanisms, the potential of OVs to inhibit the protein synthesis in tumor cells and destroy infected tumor cells by self-replication is the most probable mechanism underlying the superior efficacy of JX-594 in the reduction of *BAP1-*mutant tumors [4]. Owing to their rapid growth, *BAP1-*mutant RCC tumors require more metabolic products, which may increase their vulnerability to JX-594. However, the other mechanism of action of OVs, involving the recruitment and activation of tumor-infiltrating immune cells, should be evaluated in future studies. *BAP1* mutations may induce an increase in the number or activity of tumor-infiltrating immune cells.

Recently, Langbein et al. reported that *BAP1* maintains STING and IFN-β induction to suppress tumor growth in ccRCC [3]. This is consistent with our findings that *BAP1*-deficient tumors exhibited decreased IFN-β expression. There are no specific treatments for *BAP1*-deficient tumors in clinical practice; however, a previous study demonstrated their sensitivity to bromodomain and extraterminal (BET) inhibitors [25]. In addition to BET inhibitors, reactivating the STING-IRF3 pathway through a STING agonist has been found to be efficient at treating *BAP1*-deficient tumors [3]. Researchers have shown that BAP1 enhances the expression of IFN-β by upregulating STING-IRF3 signaling and the loss of BAP1 reduces IFN-β signaling [3]. Furthermore, reactivating the STING-IRF3 pathway through the STING agonist suppresses the growth of renal tumors in xenografts [3]. STING agonist treatment may induce tumor regression, and generate systemic immune responses, thereby inhibiting distant metastases, and inducing long-lived immunologic memory [26]. However, our findings show that *BAP1*-deficient tumors are more sensitive to JX-594 treatment than to STING agonist treatment, as demonstrated by the *BAP1*-deficient CDX model. Recent studies have demonstrated that BAP1 plays a crucial role in antiviral immunity by stabilizing STING and promoting IFN-β induction via the canonical STING–IRF3 axis. Langbein et al. showed that loss of BAP1 disrupts this pathway, attenuating IRF3-mediated type I interferon signaling [3]. Our data suggest that JX-594, a cytoplasmic DNA virus, can bypass this defect by activating IRF7, thereby restoring IFN-β production through an alternative signaling route. This IRF7-dependent compensation may underlie the enhanced therapeutic response to JX-594 observed in BAP1-deficient tumors. Consistent with this mechanism, the STING agonist diABZI, which depends on intact IRF3 signaling, was less effective in the same context. These findings are further supported by Camp et al. [27], who demonstrated that vaccinia virus infection leads to IRF7 activation and antiviral gene expression in BAP1-deficient tumor models, promoting tumor clearance despite suppressed IRF3 activity. Together, these data highlight IRF7-mediated IFN-β recovery as a mechanistic basis for the selective sensitivity of BAP1-deficient tumors to oncolytic vaccinia virus therapy.

**Fig. 6:**
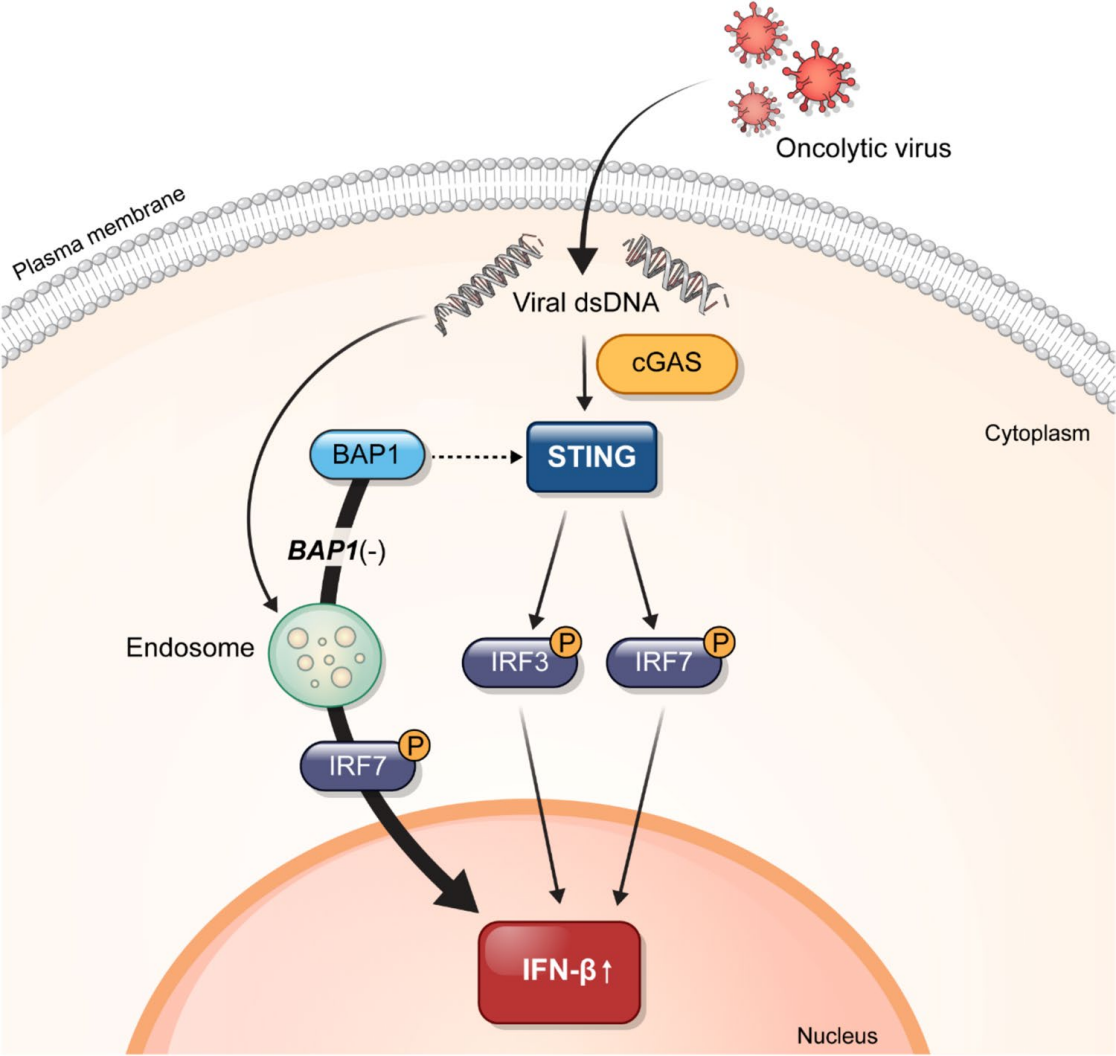
Schematic illustration of the molecular pathogenesis of oncolytic vaccinia virus

Poxvirus is a double-stranded DNA virus that replicates in the cytoplasm; it comprises variola virus, vaccinia virus (VACV), ectromelia virus, and monkeypox virus [28, 29]; JX-594 is obtained from VACV. During VACV infection, the cytosolic DNA sensor, including cyclic GMP–AMP synthase (cGAS), activates a series of downstream effectors to produce interferons, cytokines, and interleukins for an antiviral immune response [30], and increases IFN-β expression through the IRF3 pathway [31]. In addition to IRF3, IRF7 can induce positive feedback [29]. Based on our findings, we attributed the induction of IFN-β in VACV infection to the recognition of the virus by the IRF7 pathway, and not by the cGAS-STING-IRF3/IRF7 pathway (Fig. 6). *BAP1*-deficient tumors exhibited a significant increase in phosphorylated IRF7 protein levels following treatment with JX-594, rather than treatment with the STING agonist, suggesting a relationship between JX-594 and STING expression and activity in ccRCC cells.

Our findings align with a broader trend in OV development. While the phase III trial of Pexa-Vec in HCC did not meet its primary endpoint, current efforts have shifted toward combination immunotherapy and biomarker-based stratification. Active trials are investigating JX-594 in combination with cemiplimab for metastatic RCC (NCT03294083), anti-PD-L1 antibodies for melanoma (NCT04849260), and as neoadjuvant monotherapy for prostate cancer (EudraCT 2012–000704-15). Along with these trials, preclinical studies are ongoing on JX-970, a novel vaccinia virus strain engineered to improve immunogenicity and tumor targeting.

Our study identified predictive biomarkers for JX-594 treatment, but is limited to the intratumoral injection of JX-594. Given the importance of the delivery methods of therapeutics [32], our previous study demonstrated that the local and systemic injection of JX-594 facilitated adequate delivery to the primary tumor and lung metastatic sites [7]. However, to assess primary tumor sites more reliably, this study was only performed in a local injection setting. Therefore, future studies should validate these results using systemic injection settings. Another key limitation of our study is the use of nude mice, which lack functional T and NK cells. Although this allowed us to focus on tumor-intrinsic responses and the virus’s direct effects, it does not recapitulate the full immune landscape. Since one of the major therapeutic mechanisms of oncolytic viruses involves immune activation, future validation in humanized or immunocompetent syngeneic models is warranted. These models will allow for a more comprehensive assessment of JX-594-induced immune responses, particularly T cell infiltration and memory formation.

## Conclusions

Overall, this study novelly elucidated the potential of *BAP1* as a predictive biomarker for JX-594 treatment and the underlying mechanisms. Our results indicate that JX-594 suppressed *BAP1*-deficient tumor growth through IRF7-dependent IFN-β induction; reactivating this pathway may be a novel therapeutic strategy for RCC.

## Supplementary Information

Below is the link to the electronic supplementary material.

Supplementary file1 (DOCX 17 kb)

## Data Availability

No datasets were generated or analysed during the current study.

## Abbreviations

BET: Bromodomain and extraterminal;
ccRCC: Clear cell renal cell carcinoma
CDX: Cell line-derived xenograft
cGAS: Cyclic GMP–AMP synthase
EMEM: Eagle’s Minimum Essential Medium
IO: Immuno-oncology
ICI: Immune checkpoint inhibitor
mRCC: Metastatic renal cell carcinoma
OV: Oncolytic virus
RCC: Renal cell carcinoma
RPMI: Roswell park memorial institute
STING: Stimulator of interferon genes
TME: Tumor microenvironment
