# Supplementary material for: BAP1 as a predictive biomarker of therapeutic response to oncolytic vaccinia virus for metastatic renal cell carcinoma therapy
Source: Cancer Immunol Immunother. 2025 Aug 6;74(9):282. doi: 10.1007/s00262-025-04139-4 (PMC12381338; doi:10.1007/s00262-025-04139-4)
Supplement: Supplementary file 1 — Supplementary file1 (DOCX 17 kb) [file 262_2025_4139_MOESM1_ESM.docx]

Additional file

BAP1 as a Predictive Biomarker of Therapeutic Response to Oncolytic Vaccinia Virus for Metastatic Renal Cell Carcinoma Therapy

Jee Soo Park^1^, Won Sik Jang^1^, Myung Eun Lee^1^, Jongchan Kim^1,2^, Keunhee Oh^3^, Namhee Lee^3^, and Won Sik Ham^1^*

Supplementary Table 1. Polymerase chain reaction (PCR) primer sequences

|  | Primer sequences | |
| --- | --- | --- |
|  | Sense | Antisense |
| IFN-β | 5′-GCT TGG ATT CCT ACA AAG AAG CA-3′ | 5′-ATA GAT GGT CAA TGC GGC GTC-3′ |
| GAPDH | 5′-CAG CCT CAA  GAT CAT CAG CA-3′ | 5′-GGT GCT AAG  CAG TTG GTG GT-3′ |
| IFN- β: Interferon-beta | | |
